# Stability Evaluation and Stabilization of a Gastrin-Releasing Peptide Receptor (GRPR) Targeting Imaging Pharmaceutical

**DOI:** 10.3390/molecules24162878

**Published:** 2019-08-08

**Authors:** Arijit Ghosh, Karen Woolum, Shankaran Kothandaraman, Michael F. Tweedle, Krishan Kumar

**Affiliations:** Laboratory for Translational Research in Imaging Pharmaceuticals, Wright Center of Innovation in Biomedical Imaging, Department of Radiology, The Ohio State University, Columbus, OH 43212, USA

**Keywords:** gastrin-releasing peptide receptor, GRPR, acetate buffer stability, in-vitro stability, mouse serum stability, canine serum stability, human serum stability, imaging pharmaceuticals, radiotracers, radiolabeling

## Abstract

The prostate-specific membrane antigen (PSMA) and gastrin-releasing peptide receptor (GRPR) are identified as important targets on prostate cancer. Receptor-targeting radiolabeled imaging pharmaceuticals with high affinity and specificity are useful in studying and monitoring biological processes and responses. Two potential imaging pharmaceuticals, AMBA agonist (where AMBA = DO3A-CH2CO-G-[4-aminobenzyl]- Gln-Trp-Ala-Val-Gly-His-Leu-Met-NH_2_) and RM1 antagonist (where RM1 = DO3A-CH_2_CO-G-[4-aminobenzyl]-D-Phe-Gln-Trp-Ala-Val-Gly-His-Sta-Leu-NH_2_), have demonstrated high binding affinity (IC_50_) to GRP receptors and high tumor uptake. Antagonists, despite the poor tumor cell internalization properties, can show clearer images and pharmacokinetic profiles by virtue of their higher tumor uptake in animal models compared to agonists. For characterization, development, and translation of a potential imaging pharmaceutical into the clinic, it must be evaluated in a series of tests, including in vitro cell binding assays, in vitro buffer and serum stability studies, the biodistribution of the radiolabeled material, and finally imaging studies in preclinical animal models. Data related to acetate buffer, mouse, canine, and human sera stability of ^177^Lu-labeled RM1 are presented here and compared with the acetate buffer and sera stability data of AMBA agonist. The samples of ^177^Lu-labeled RM1 with a high radioconcentration degrade faster than low-radioconcentration samples upon storage at 2–8 °C. Addition of stabilizers, ascorbic acid and gentisic acid, improve the stability of ^177^Lu-labeled RM1 significantly with gentisic acid being more efficient than ascorbic acid as a stabilizer. The degradation kinetics of ^177^Lu-labeled AMBA and RM1 in sera follow the order (fastest to slowest): mouse > canine > human sera. Finally, ^177^Lu-labeled RM1 antagonist is slower to degrade in mouse, canine, and human sera than ^177^Lu-labeled AMBA agonist, further suggesting that an antagonist is a more promising candidate than agonist for the positron emission tomography (PET) imaging and therapy of prostate cancer patients.

## 1. Introduction 

Prostate cancer (PCa), other than skin cancer, is the most common cancer and is the second leading cause of cancer deaths, behind lung cancer, among men in the United States [1]. The American Cancer Society estimates about 174,650 new cases of prostate cancer and about 31,620 deaths from this disease in 2019. It is also estimated that about 1 in 9 men will be diagnosed with prostate cancer during their lifetime. Therefore, early detection of primary disease and its metastases is critical for clinical staging, prognosis, and therapy management. 

Currently, prostate cancer is diagnosed in early stages by elevated serum prostate-specific antigen (PSA) levels, by clinical symptoms, and by imaging techniques such as magnetic resonance imaging (MRI), computed tomography (CT) and transrectal ultrasound, and finally confirmed by prostate biopsy. However, PSA levels can be elevated in benign conditions, and cancer lesions may be missed by imaging. Since the prostate cancer metastasizes to bones, single-photon emission computed tomography (SPECT) bone scintigraphy using ^99m^Tc-labeled methylene diphosphate (^99m^Tc-MDP) has been used for the past several decades [2]. However, this technique is limited because of its low specificity and often requires correlation of the results with other supplemental modalities. 

Recently, positron emission tomography (PET) using imaging pharmaceuticals and in combination with other modalities has been found useful in the detection of biochemical relapse, recurrence, and extent of metastatic disease. Several imaging pharmaceuticals have been proposed for molecular imaging of prostate cancer, including ^18^F-NaF (^18^F-sodium fluoride) for bone imaging due to prostate cancer metastasis, choline (^11^C-Choline and ^18^F-Choline) as a marker of membrane cell proliferation, incorporation of ^11^C-Acetate into intracellular phosphatidylcholine membrane, and ^18^F-FACBC (^18^F-fluciclovine; 1-amino-3-fluorocyclo-butane-1-carboxylic acid) to monitor amino acid transport [3,4,5]. Numerous studies, however, have been reported suggesting limited sensitivity and specificity of these imaging pharmaceuticals in detecting prostate cancer in patients with low PSA levels [6].

The need for receptor-targeted imaging pharmaceuticals has led to the discovery and development of numerous radiolabeled peptides and proteins that can target receptors that are known to overexpress on certain tumors [7,8,9]. Two such receptors are identified in prostate cancer cells: prostate-specific membrane antigen (PSMA) and the gastrin-releasing peptide receptor (GRPR).

PSMA is a transmembrane protein that has significantly elevated expression in prostate cancer cells than in the benign prostatic tissues. Several PSMA-targeted PET imaging pharmaceuticals have been developed and evaluated in the past in preclinical and clinical environments [10,11,12,13]. This includes ^68^Ga- and ^18^F-AlF-labeled PSMA-11, PSMA-617, PSMA I&T, THP-PSMA, ^18^F-labeled DCFBC, DCFPyL, PSMA-1007, ^99m^Tc-labeled MIP-1404, ^123^I- and ^124^I-labeled MIP-1095, ^18^F-AlF-labeled NOTA-DUPA-Pep, and other HBED analogs. 

GRPR, a subtype of the bombesin receptor family, is an attractive target for imaging tumors with neuroendocrine origin including prostate, breast, and small cell lung cancers. For prostate cancer, especially, high-affinity GRPR expression has been identified in tissue biopsy samples and immortalized cell lines [14,15]. Bombesin (BBN) is a 14 amino acid amphibian peptide analog of the 27 amino acid mammalian GRP. BBN and GRP share a homologous seven amino acid amidated C-terminus, Trp-Ala-Val-Gly-His-Leu-Met-NH_2_, which is necessary for binding as an agonist to the GRPR [16]. Synthetic BBNs are modified versions of the above peptide sharing the common seven amino acid C-terminus. The N-terminus is free for conjugation with appropriate radiolabeled metal chelate for various applications. 

**Figure 1:**
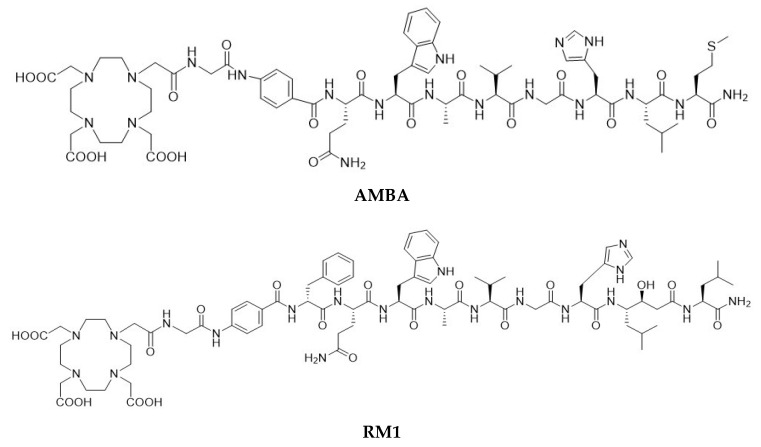
Structure of DO3A-CH2CO-G-[4-aminobenzyl]- Gln-Trp-Ala-Val-Gly-His-Leu-Met-NH_2_ (AMBA) and DO3A-CH_2_CO-G-[4-aminobenzyl]- D-Phe-Gln-Trp-Ala-Val-Gly-His-Sta-Leu-NH_2_ (RM1).

The first proof-of-concept GRPR targeting study was conducted using a ^99m^Tc-labeled BBN (7–14) conjugate (RP527) for breast and prostate cancer imaging [17]. Among various other BBN conjugates [18,19,20,21,22], a BBN-like agonist peptide—AMBA (where AMBA = DO3A-CH_2_CO-G[4-aminiobenzyl]-Gln-Trp-Ala-Val-Gly-His-Leu-Met-NH_2_, Figure 1)—labeled with ^177^Lu showed high affinity towards the gastrin-releasing peptide (GRP) receptors in a PC-3 cell line (measured IC_50_ values were 4.75 ± 0.25 and 2.5 ± 0.5 nM for AMBA and Lu(AMBA), respectively [23,24]). Despite the general consensus related to the high tumor cell internalization rate of agonists for optimal imaging and in vivo therapy [25], it has been recently shown that high-affinity somatostatin antagonists (both sst_2_- and sst_3_-selective analogs), despite their poor tumor cell internalization properties, can show clearer images and pharmacokinetic profiles [26]. These observations led to a change in focus towards the discovery and development of GRPR antagonists for cancer imaging and therapy [27].

Several classes of BBN antagonists have been designed by modifying the C-terminal residues of naturally amidated BBN agonists [28,29,30,31]. A series of potent radiolabeled antagonists, ^111^In- and ^68^Ga- labeled RM1 (where RM1 = DO3A-CH_2_CO-G-[4-aminobenzyl]- D-Phe-Gln-Trp-Ala-Val-Gly-His-Sta-Leu-NH_2_, where Sta is a statyl residue Figure 1) and ^111^In-labeled RM2 peptides, showed higher tumor uptake and better pharmacokinetics than the corresponding agonists [32,33]. Additionally, ^68^Ga-, ^111^In-, and ^18^F-AlF-labeled NOTA-PEG_2_-RM26 (where RM26 = D-Phe-Gln-Trp-Ala-Val-Gly-His-Sta-Leu-NH_2_) conjugates demonstrated favorable properties for cell binding and internalization (IC_50_ = 1.24 ± 0.29 nM for ^111^In- and 0.91 ± 0.19 nM for ^68^Ga-labeled NOTA- PEG_2_-RM26), specific tumor uptake (8.1 ± 0.4%ID/g for ^68^Ga- and 5.7 ± 0.3%ID/g for ^111^In-labeled NOTA- PEG_2_-RM26), and imaging of GRPR-expression in PC-3 tumor-bearing mice [34,35]. A ^68^Ga-labeled GRPR antagonist (NOTA-PEG_3_-RM26) was also shown to be a more promising candidate than a ^68^Ga-labeled agonist (NOTA-Aca-BBN_7-14_) for imaging of prostate cancer [36]. 

The GRPR targeting imaging pharmaceutical, RM1, is an attractive theranostic choice as the DO3A-CH_2_CO- moiety forms stable chelates with trivalent metal ions, ^68^Ga, ^111^In, and ^177^Lu. For characterization, development, and translation of a potential imaging pharmaceutical into the clinic, it must be evaluated in a series of tests, including in vitro cell binding assays, buffer and serum stability studies, the biodistribution of the radiolabeled material, and finally imaging studies in preclinical animal models [37]. In vitro cell binding, biodistribution, and imaging studies in preclinical models were reported for the two potential imaging pharmaceuticals (i.e., ^177^Lu- and ^68^Ga-labeled AMBA [23,24,38] and ^111^In- and ^68^Ga-labeled RM1 [32]). Additionally, the feasibility and safety of ^68^Ga-labeled RM1 were tested in humans [39]. Interestingly, there are no significant changes in the biological and stability properties of the agonist or antagonist by changing the metallic radionuclide in the imaging pharmaceutical. The in vitro mouse and human sera stability of ^177^Lu-labeled AMBA were reported previously [23]; however, there are no data available related to mouse, canine, and human sera stability of ^177^Lu-labeled RM1 and canine serum stability of ^177^Lu-labeled AMBA. Determination of canine serum stability of ^177^Lu-labeled RM1 is important from the perspective that we have recently developed a canine prostate tumor model for evaluation of the GRPR receptor-specific peptides [40] for cancer imaging and peptide receptor radiotherapy (PRRT). 

The objectives of the present work were to study (1) in vitro stability of ^177^Lu-labeled RM1 (Antagonist) and AMBA (agonist) in acetate buffer with low (0.037–0.056 GBq/mL) radioconcentrations stored at room temperature and at 2–8 °C, (2) in vitro stability of ^177^Lu-labeled RM1 with high (~0.74 GBq/mL) radioconcentrations under 2–8 °C storage conditions (3) the effect of stabilizers, ascorbic acid, and gentisic acid, on the in vitro stability of high radioconcentration, acetate buffered, ^177^Lu-labeled RM1 samples stored at 2–8 °C, (4) in vitro stability of ^177^Lu-labeled RM1 and AMBA in mouse, canine, and human sera and canine serum, respectively , and (5 to compare the in vitro stability of ^177^Lu-labeled RM1 and AMBA in acetate buffer and mouse, canine, and human sera. 

For this purpose, ^177^Lu radionuclide (with 6.7 d t_1/2_ and 113 keV gamma peak) than ^68^Ga (with 68 min half-life) was used as a radiotracer of choice to monitor the degradation of ^177^Lu-labeled AMBA and RM1 in acetate buffer, mouse, canine, and human sera. Because of the longer half-life of ^177^Lu, a single-labeled AMBA and RM1 could be used for multiple experiments leading to less variation in the data due to batch-to-batch variability of the radiolabeled materials. 

We report herein our results related to (1) in vitro stability of low- radioconcentration samples of ^177^Lu-labeled RM1 and AMBA upon storage at room temperature and 2–8 °C and high-radioconcentration samples of ^177^Lu-labeled RM1 upon storage at 2–8 °C, (2) effect of stabilizers, ascorbic acid, and gentisic acid on the stability of high-radioconcentration samples of ^177^Lu-labeled RM1, (3) the comparison of half-lives of degradation of ^177^Lu-labeled AMBA and RM1, in acetate buffer, and (4) comparison of degradation kinetics of ^177^Lu-labeled RM1 antagonist and AMBA agonist in mouse, canine, and human sera. 

## 2. Results and Discussion 

### 2.1. Characterization of RM1, AMBA, Lu-RM1, and Lu-AMBA

The HPLC analysis of RM1 and AMBA and their lutetium chelates demonstrated >94% purity (Figures S1–S4). The MALDI-TOF mass spectral analysis (Figures S5–S8) gave m/e peaks for m + H of the compound (calculated m/e for molecular mass in the parenthesis) as follows: 1675.71 (1674.66) RM1; 1502.73 (1501.71) AMBA; 1847.67 (1846.77) Lu-RM1; and 1674.56 (1673.68) Lu-AMBA. Based on the retention times under similar HPLC conditions (Figures S3 and S4), it can be concluded that the Lu-RM1 was slightly more lipophilic than Lu-AMBA, which was in good agreement with the log D measurements. The log D values (average of n = 3 with standard deviation in the parenthesis) determined were −2.46 (0.07) and −2.57 (0.04) for Lu-RM1 and Lu-AMBA, respectively. 

### 2.2. Characterization of ^177^Lu-Labeled RM1 and AMBA 

^177^Lu-labeled AMBA and RM1 were analyzed for radioactivity concentration and radiochemical purity (RCP) by using a dose calibrator and a reversed-phase high-performance liquid chromatography (RP-HPLC) method, respectively. The retention times (min), the radio concentration (GBq/mL), the molar activity (GBq/mmol), and the radiochemical purity (%RCP) were: ^177^Lu-labeled AMBA 13.9–14.0 min, 0.034 GBq/mL, 617 GBq/mmol, >99% and ^177^Lu-labeled RM1 14.9–15.2 min, 0.034 GBq/mL, 699.3 GBq/mmol, >99%, respectively. The radiochemical yield of ^177^Lu labeling of RM1 or AMBA was >99% as confirmed by the RCP data. Figures S9 and S10 show chromatograms of Lu/^177^Lu-labeled RM1 and AMBA, respectively. 

### 2.3. Stability of Low-Radioconcentration Samples of ^177^Lu-Labeled RM1 and AMBA in Acetate Buffer

The final pH of the ^177^Lu-labeled RM1 and AMBA, post radiolabeling, was usually ~5 to 5.5, and the acetate buffer concentration was 0.1 M. Consequently, the degradation kinetics of ^177^Lu-labeled RM1 and AMBA were evaluated in this environment after storage at room temperature (25 °C) and 2–8 °C for 5 d. Both samples were analyzed using an RP-HPLC method at various time points. 

**Figure 2:**
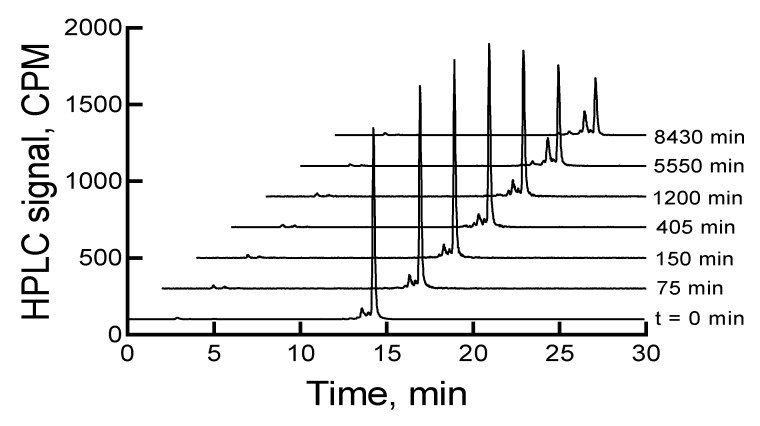
HPLC chromatograms of ^177^Lu-labeled RM1 in pH 5 acetate buffer (0.1 M) stored at 2–8 °C as a function of time. CPM is counts per minute.

The HPLC analysis of ^177^Lu-labeled RM1, immediately after completion of the radiolabeling experiment, showed a major peak at ~15 min (~88.3%) in the HPLC chromatogram. There were three other minor peaks (percentages are given in the parenthesis) at 2.9 (1.0), 14.3 (~7), and 14.7 (3.6) min. When the samples were left at room temperature or 2–8 °C storage conditions, the main peak related to ^177^Lu-labeled RM1 decreased, and the 14.3 min peak increased. In almost 6 d, the main peaks decreased from 88.3% to 49% and 52%, and the 14.3 min peaks increased to 30% and 25% under room temperature and 2–8 °C storage conditions, respectively. Figure 2 shows HPLC chromatograms of a ^177^Lu-labeled RM1 as a function of time for a sample stored at 2–8 °C. There were some other minor peaks (~1% to 4%) that eluted at lower retention times. Similar degradation patterns were observed for ^177^Lu-labeled AMBA; however, the AMBA agonist degraded at a relatively faster rate (i.e., 70% and 60% degradation upon storage at room temperature and 2–8 °C for 6 d, respectively). Additionally, the major degradant of ^177^Lu-labeled AMBA eluted at an earlier time than the major degradant of ^177^Lu-labeled RM1, suggesting that the AMBA degradant was probably more hydrophilic than the degradant of RM1. 

**Figure 3:**
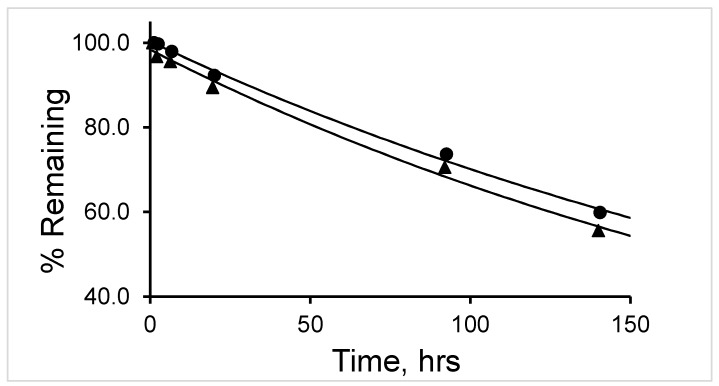
A plot of percent remaining of ^177^Lu-Labeled RM1 pH 5 acetate buffer (0.1 M) stored at 2–8 °C (circles) and room temperature (triangles) as a function of time.

The percent remaining of ^177^Lu-labeled RM1 was fitted into a first-order kinetics model (Figure 3). The average (with standard deviation in the parenthesis for n = 3) t_1/2_ (half-life) values for ^177^Lu-labeled RM1 and AMBA were calculated as 164.3 (3.6) and 195.6 (3.6) h (~7 and ~8 d) (Table 1) and 87 (3.9) and 127.7 (2.1) h (~4 and ~5 d) at room temperature and 2–8 °C storage conditions, respectively. In summary, the ^177^Lu-labeled RM1 (antagonist) was found to degrade slower than the ^177^Lu-labeled AMBA (agonist) in both storage conditions. 

### 2.4. Stability Evaluation and Stabilization of High-Radioconcentration ^177^Lu-Labeled RM1 Samples by Ascorbic Acid and Gentisic Acid

The ^177^Lu-labeled RM1 samples in acetate buffer, with 0.74 GBq/mL radio concentration, 10,175–12,765 GBq/mmol molar activity, and >99% RCP, were prepared for high-radioconcentration sample stability studies. The samples, when stored at 2–8 °C, degraded significantly faster than the samples with low radioconcentrations due to the radiolytic and oxidative degradation of the peptide moiety by a high level of radioactivity. For example, complete degradation of ^177^Lu-labeled RM1 was seen after ~3 d with several early eluting peaks in the HPLC, with a major peak being at 12.8 min with 46% peak area. From a time-dependent study, an average (with standard deviation in the parenthesis for n = 3) half-life of degradation of ^177^Lu-labeled RM1 was calculated as ~8.5 (0.2) h. Degradation of the high-radioconcentration sample in acetate buffer was at least 20 times faster than the degradation of the low-radioconcentration samples of ^177^Lu-labeled RM1.

Traditionally, ascorbic acid and gentisic acid are known as radio stabilizers. Consequently, we investigated the effect of these two stabilizers on the stability of ^177^Lu-labeled RM1. An improvement in the stability of the high-radioconcentration sample of ^177^Lu-labeled RM1 was observed in the presence of 500 µg ascorbic acid when stored at 2–8 °C. For example, the percentages of ^177^Lu-labeled RM1 degraded were 9.7%, 21.5%, 34.8%, 87.1%, 89.5%, and 92% at 22, 46, 70, 118, 142, and 166 h, respectively. Several peaks (percentages given in parentheses) appeared at 12.3 (9%), 12.9 (19.9%), and 13.7 (43%) min after 166 h storage at 2–8 °C. Surprisingly, the peak eluting at 2.9 min corresponding to free ^177^Lu/Lu increased to 12.8% over the period of 7 d, even though the pH of the sample was constant throughout the study. A small amount (2.4%–3.5%) of the possible ^177^Lu(DO3A-amide) moiety was observed during the later days as well. An average (with standard deviation in the parenthesis for n = 3) half-life of degradation was calculated as 39.2 (0.6) h (~1.6 d) by fitting the percent remained versus time data into a first-order kinetics model. From the comparison of the control experiment, it can be concluded that the degradation of a high-radioconcentration sample of ^177^Lu-labeled RM1 was reduced at least by a factor of 5 in the presence of ascorbic acid. 

**Figure 4:**
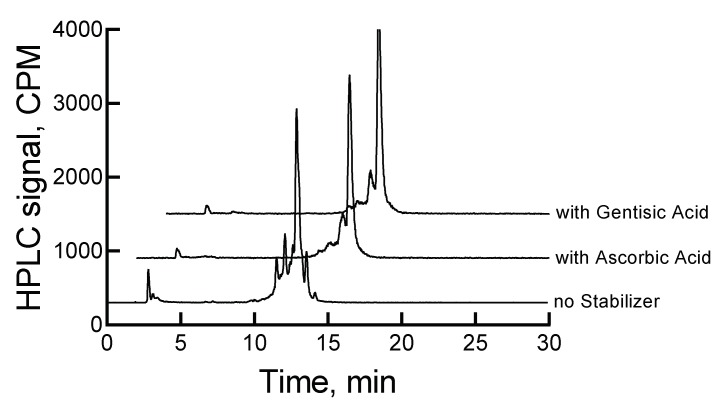
HPLC chromatograms for ^177^Lu-labeled RM1 in 0.1 M acetate buffer, pH 5 at 2–8 °C storage in the absence and presence of stabilizers after 46 h.

A similar amount of gentisic acid (500 µg) was found to be more effective in preventing the radiolytic degradation of ^177^Lu-labeled RM1. For example, 3.9%, 13.3%, 18.3%, 32.2%, 36%, and 36.8% degradations at 22, 46, 70, 118, 142, and 166 h were observed. After 7 d storage of the mixture of ^177^Lu-labeled RM1 and gentisic acid, major degradation peaks seen were at 13.7 min (23.2%), 2.9 min (~6%) due to free ^177^Lu/Lu, and 3.2 min (~2%) due to ^177^Lu(DO3A-amide). Figure 4 shows the HPLC chromatograms for ^177^Lu-labeled RM1 in the absence and presence of stabilizers at 46 h.

An average (with standard deviation in the parenthesis for n = 3) t_1/2_ for degradation of ^177^Lu-labeled RM1 in the presence of 500 µg gentisic acid was calculated as 142.5 (1.4) h (~6 d), which was almost 17-fold improvement of the stability of ^177^Lu-labeled RM1 than without any stabilizer. In summary, gentisic acid appears to be more effective than ascorbic acid in retarding the degradation kinetics due to radiolysis and stabilizing RM1. Table 1 provides a summary of the stability of low- and high-radioconcentration samples of ^177^Lu-labeled RM1 with and without stabilizers.

### 2.5. Stability of ^177^Lu-Labeled RM1 and AMBA in Mouse, Canine, and Human Sera 

For mouse, canine, and human sera stability studies, 50 µL of ^177^Lu-labeled RM1 or AMBA was mixed with 450 µL of mouse or canine or human serum. The mixtures were incubated at 37 °C for 24 h, samples were processed at each time point, and analyzed by an RP-HPLC method. 

**Figure 5:**
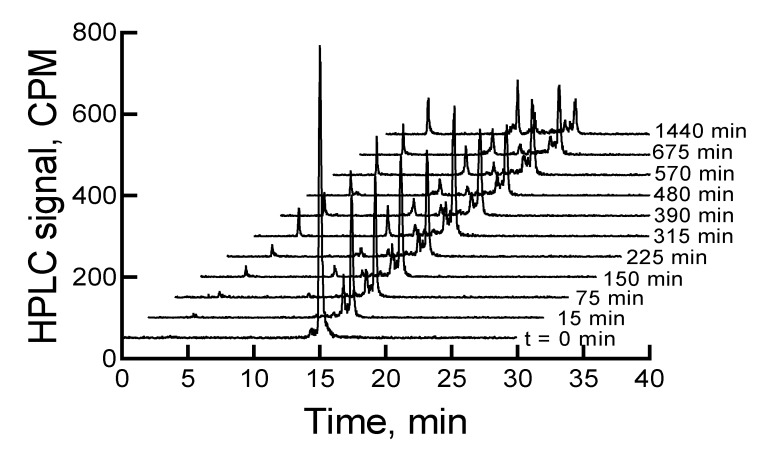
HPLC chromatograms of ^177^Lu-labeled RM1 in mouse serum at 37 °C as a function of incubation time over 24 h.

The ^177^Lu-labeled RM1 degraded in mouse serum (expressed as percent remaining) with time, that is, 91.9, 85.3, 78.7, 73.6, 71.1, 69.6, 61.7, and 59.1 after 75, 150, 225, 315, 390, 480, 570, and 675 min incubation at 37 °C (Figure 5). On the contrary, only 10% to 12% of ^177^Lu-labeled RM1 was degraded within the same time period in pH 5 sodium acetate buffer environment when it was stored at room temperature or a 2–8 °C storage condition. In the mouse serum incubated ^177^Lu-labeled RM1 samples, new peaks appeared at lower retention times of 3.4, 10.1, 11.7, 12.3, and 12.9 min (or 0.22, 0.66, 0.77, 0.81, and 0.85 relative retention times (RRTs)). With the exception of the degradant with 0.77 and 0.85 RRTs, which remained unchanged, the percent of other degradant generally increased, suggesting the formation of less lipophilic metabolites of ^177^Lu-labeled RM1. After 1440 min of incubation in mouse serum, complete degradation of the ^177^Lu-labeled RM1 was observed with three major peaks eluting at 3.3, 10.0, and 14.4 min with areas of 17.1%, 29%, and 22%, respectively.

The early eluting peak at 3.4 min most likely is the ^177^Lu(DO3A-amide) moiety, since we have established that free ^177^Lu/^177^Lu-EDTA elutes at 2.9 min under these HPLC conditions. Furthermore, this is also consistent with our previously published in vitro serum stability of ^153^Gd-labeled DO3A conjugated peptides [37]. 

**Figure 6:**
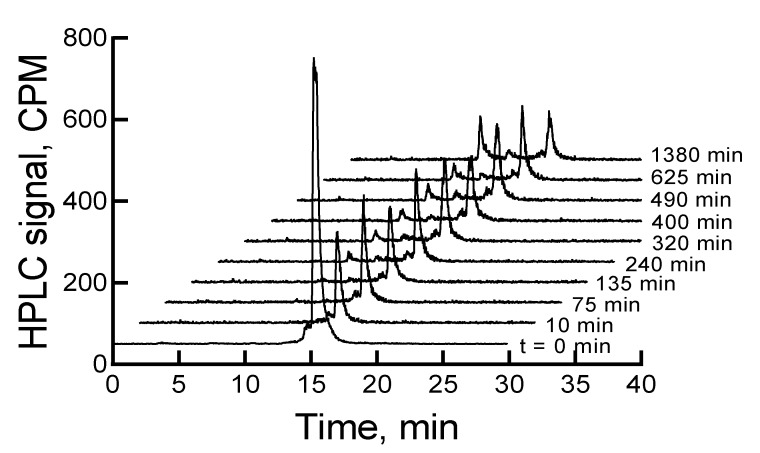
HPLC chromatograms of ^177^Lu-labeled RM1 in canine serum at 37 °C as a function of incubation time over 24 h.

When the mixture of canine serum and ^177^Lu-labeled RM1 or AMBA was incubated for 75, 135, 240, 320, 400, 490, 625, and 1380 min, the ^177^Lu-labeled RM1 degraded with time. The percent remaining was calculated from the RP-HPLC analysis of ^177^Lu-labeled RM1 as 99.98, 94.6, 87.2, 85.3, 82.5, 77.8, 75.0, 53.5 at 75, 135, 240, 320, 400, 490, 625, and 1380 min, respectively (Figure 6). The degradants in canine serum were similar to the degradants observed in mouse serum eluting at 10.1, 12.3, and 12.9 min. The ^177^Lu-labeled AMBA degraded in canine serum faster than ^177^Lu-labeled RM1. For example, the percent remaining of ^177^Lu-labeled AMBA were 89.5, 80.7, 75.0, 70.9, 66.5, 62.1, 55, and 18.4 at 75, 135, 240, 320, 400, 490, 625, and 1380 min, respectively. The peaks at lower retention times appeared at 3.2, 6.2, 9.4, 11, and 12.1 min and showed a gradual increase with longer incubation of the samples. 

The ^177^Lu-labeled RM1 and human serum mixture samples were incubated for 24 h, and samples were analyzed using RP-HPLC. Contrary to the mouse and canine sera studies, slow degradation of ^177^Lu-labeled RM1 (<10%) was observed in human serum in 24 h. However, the metabolites eluting at 3.4, 10.1, and 12.2 min (although lower percentages) were observed to appear at later time points of incubation. It is interesting to note that the metabolites in the mouse, canine, and human sera samples had similar properties, suggesting that the degradation of ^177^Lu-labeled RM1 follows common degradation pathways/steps independent of the source of the serum. Most of the radioactivity was in the form of bound ^177^Lu either to the peptide fragment conjugated DO3A or DO3A-amide. No significant amount of free ^177^Lu was detected in the serum stability studies.

**Figure 7:**
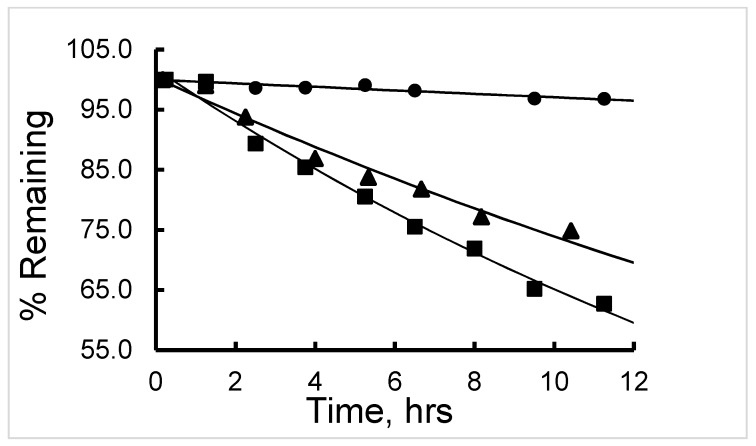
A plot of percent remaining of ^177^Lu-Labeled RM1, in human (circles), canine (triangles), and mouse sera (squares) at 37 °C, as a function of time.

The half-life (t_1/2_) of degradation of ^177^Lu-labeled RM1 (in mouse, canine, and human sera) and ^177^Lu-labeled AMBA (in canine serum) were calculated by fitting the percent remaining of ^177^Lu-labeled peptide versus incubation time data into a first-order kinetics model (Figure 7). Average (with standard deviation in the parenthesis for n = 3) t_1/2_ (in h) values for degradation of ^177^Lu-labeled RM1 were calculated as 16.4 (0.6), 18.9 (0.8), and 141 (5) h (Table 2) in mouse, canine and human sera, respectively. An average (with standard deviation in the parenthesis for n = 3) t_1/2_ (in h) for degradation of ^177^Lu-labeled AMBA in canine serum was determined as 10.1 (0.5) h in the present work, and half-lives of degradation in mouse and human sera were reported as 3.1 and 38.8 h, respectively [23,24]. In summary, (1) the half-life of degradation (stability) of ^177^Lu-labeled RM1 and AMBA followed the order: human > canine > ~mouse, and more interestingly, (2) in general, the antagonist, ^177^Lu-labeled RM1, was slower to degrade than the agonist, ^177^Lu-labeled AMBA, in mouse, canine, and human sera.

Determination of serum stability of a new chemical entity (NCE), such as a peptide or a small molecule containing amide bonds, is an important step in the development of a potential imaging pharmaceutical. Various esterases appear to have the most significant effect on drugs circulating in the serum [41]. Carboxylesterases that will hydrolyze ester or amide groups in peptides or small molecules have not been detected in human, dog, or monkey plasma. However, rats, rabbits, and other small animals possess this class of esterases, which may be responsible for faster degradation of ^177^Lu-labeled RM1 and AMBA in mouse serum. Faster degradation of ^177^Lu-labeled AMBA than RM1 may be due to the presence of methionine amino acid in the former. It was found previously that methionine residue in AMBA is readily oxidized to its biologically inactive methionine sulfoxide (Met(O)), forming ^177^Lu-AMBA-Met(O) [42].

## 3. Materials and Methods

### 3.1. General 

Commercial mouse and human serum from MP Biomedicals (Solon, OH, USA) and canine serum from Immunoreagents, Inc. (Raleigh, NC, USA) were used in the in vitro serum stability studies. Buffers were prepared, and pH was controlled using sodium acetate (Sigma Aldrich, St. Louis, MO, USA), sodium hydroxide, and hydrochloric acid (Fisher Scientific, Fair Lawn, NJ, USA). Acetonitrile and Tris acetate containing ethylenediaminetetraacetic acid (EDTA) (all from Fisher) were used for mobile phase preparations. Ascorbic acid (Sigma Aldrich) and gentisic acid (Acros Organics, Molinons, France, distributed by Fisher Scientific) were used as stabilizers. Octanol (Sigma Aldrich, St. Louis, MO, USA) was used for log D determinations. Absolute ethanol for the precipitation of serum proteins in the degradation kinetics/stability experiments was purchased from Fisher Scientific. A sample of ^177^lutetium chloride (^177^LuCl_3_) in 0.05 M hydrochloric acid (molar activity > 555 GBq/mg Lu, produced by Missouri University Research Reactor, MURR, Columba, MO, USA) was supplied by Oak Ridge, TN, USA for radiolabeling of RM1 and AMBA. A block heater (Labnet International, Edison, NJ, USA) was used for controlling temperature of the reaction of ^177^LuCl_3_ with AMBA or RM1 and the maintenance of incubation temperature of ^177^Lu-labeled AMBA or RM1 and serum mixtures. All pH measurements were made with a combination glass electrode and a model S220 pH/Ion meter (Mettler Toledo, Columbus, OH, USA). Deionized MilliQ water (Millipore Sigma, Burlington, MA, USA) was used for all solution preparations.

### 3.2. Chemistry

Two GRPR targeting ligands, AMBA (agonist, a well-characterized gift from Bracco Diagnostics, Inc., Monroe Township, NJ, USA) and RM1 (antagonist, synthesized and purified by a modified procedure described elsewhere [32]) as well as Lu-AMBA (a well-characterized gift from Bracco Diagnostics, Inc.,) and Lu-RM1 (obtained from in-situ synthesis) were analyzed by RP-HPLC and characterized by MALDI/TOF mass spectrometry. For the in situ synthesis of Lu-RM1, 1 mg (0.597 μmol) of an HPLC purified RM1 was dissolved in 1 mL 0.1 M sodium acetate buffer (pH of 4). The peptide solution was then mixed with a slight excess of lutetium acetate in 0.05 M HCl. The reaction mixture was heated at 95 °C in a heating block for 20–30 min. The pH of the reaction mixture was then slowly raised to ~ 5.5 (checked with a pH paper) using 0.1 M sodium acetate solution (pH of 7) with intermittent heating at 95 °C for 10–15 min. The final volume of the reaction mixture was made up to ~1 mL. 

### 3.3. Radiochemistry

The ^177^Lu-labeled AMBA and RM1 were prepared by a standard procedure developed in our laboratories. In a typical experiment, a stock sample solution (1 mg/mL) of an HPLC purified AMBA or RM1 was prepared by dissolving an appropriate amount of the peptide chelating agent conjugate in 0.1 M sodium acetate buffer (pH of 4). A sample of a desired amount of radioactivity (^177^LuCl_3_) in 0.05 M HCl was then mixed with an excess (typically 10%–20%) of AMBA or RM1 (50 to 125 µg) in the acetate buffer medium. The reaction mixture was heated at 95 °C in the heating block for 20–30 min. The pH of the reaction mixture was then slowly raised to ~5.0 to 5.5 (checked with a pH paper) using 0.1 M sodium acetate solution with intermittent heating at 95 °C for 10–15 min. 

### 3.4. Analytics

A dose calibrator (Capintec, Florham Park, NJ, USA) and a Wizard 2480 gamma counter (Perkin Elmer, Waltham, MA, USA) were used for radioactivity measurements in the samples. A model 1100 Agilent HPLC system (Agilent, Wilmington, DE, USA) was used for the analysis of ^177^Lu-labeled RM1 and AMBA and stability samples. The system consisted of a quaternary pump, degasser, column compartment capable of controlling the temperature at ambient or at 37 °C, auto-injector, a multiwavelength detector, and a Flow Ram radioisotope detector (Lab Logic, Sheffield, UK). The system was controlled by Laura software from Lab Logic. A Waters (Milford, MA, USA) Zorbax Bonus RP C_18_ column (4.6 × 250 mm, 5 µm) was used for the analysis of ^177^Lu-labeled AMBA and RM1 samples and for the separation of the ^177^Lu-labeled AMBA and RM1 from their in vitro metabolic degradants in mouse, human, and canine sera stability samples. 

All mass spectral analyses were performed at the Campus Chemical Instrument Center of the Ohio State University (Columbus, OH, USA) using a Bruker UltrafleXtreme MALDI-TOF MS system. An RP-HPLC method, involving a gradient mobile phase system of 50 mM Tris acetate, 10 mM EDTA at pH 7.2 (solvent A), and acetonitrile (solvent B) with 1 mL/min flow rate, was used to monitor the extent of the reaction of ^177^Lu/Lu with AMBA and RM1; for the purity analysis of RM1, AMBA, Lu-RM1, Lu-AMBA, and of the final ^177^Lu-labeled samples; and for monitoring the degradation of ^177^Lu-labeled RM1 or AMBA samples in acetate buffer, mouse, canine, and human sera. The EDTA was incorporated to detect any unreacted ^177^Lu/Lu in the samples. The gradient program used was 0% B at the beginning, raising the percentage of B to 50% in 15 min, holding B at 50% for 4 min, followed by decreasing the percentage of B to 0% in 5 min and continuing 0% for an additional 9 min. The total run time was 30 min.

The log D values were determined by a traditional shake-flask method. In a typical experiment, 10 μL sample of ^177^Lu-labeled AMBA or RM1 with 0.037–0.052 GBq/mL radioconcentration and 814 GBq/mmol molar activity were added to a 4 mL mixture of 50:50 octanol:water. The mixture was shaken for one minute and mixed well. The sample was stored at room temperature for 5 min for separation of the two layers. Sample from each layer (25 μL) was added to a counting tube containing 250 μL water. The samples were mixed and counted. Triplicate measurements were made. The log D values were calculated using Equation (1):

D = Counts in organic phase/Counts in the aqueous phase.(1)

### 3.5. Stability

All samples of ^177^Lu-labeled RM1 were prepared in 0.1 M acetate buffer, pH 5–5.5. The ^177^Lu-labeled RM1 samples, with a low radioconcentration, were stored at room temperature and at 2–8 °C storage conditions for 6 d for stability studies. The samples with a high radioconcentration, without and with a stabilizer (gentisic acid or ascorbic acid), were stored 2–8 °C for 2 and 7 d, respectively. For the effect of stabilizer (ascorbic acid or gentisic acid) on the stability of the high-radioconcentration samples of ^177^Lu-labeled RM1, the samples were mixed with 500 µg of ascorbic or gentisic acid post radiolabeling. The stability or degradation of the samples was monitored by a reduction in the percent HPLC peak area of ^177^Lu-labeled RM1 with time. 

A literature procedure was followed for the serum stability studies of ^177^Lu-labeled RM1 and AMBA [37,43]. On the day of the experiment, a small portion of the frozen mouse, human or canine serum was thawed at room temperature. A stock sample of ^177^Lu-labeled RM1 or AMBA and the serum were equilibrated at 37 °C in the block heater initially. Several mixtures, containing 50 µL of ^177^Lu-labeled RM1 or AMBA (usually 1.48 to 2.22 MBq radioactivity) and 450 µL of the mouse or human or canine serum, were prepared in HPLC vials and incubated at 37 °C in the block heater. At a selected time point (typically 15, 75, 150, 225, 315, 390, 480, 570, 675, and 1440 min), a vial was taken out from the heating block, and degradation of the ^177^Lu-labeled AMBA or RM1 was stopped by adding cold (4 °C) absolute ethanol (500 µL). The sample was then centrifuged at 6000 rpm for 15 min, and 200 µL of the supernatant solution was isolated and analyzed by an RP- HPLC method given above. A control experiment was conducted where samples of ^177^Lu-labeled AMBA and RM1 were incubated in the water at 37 °C and analyzed by the RP-HPLC method. 

Degradation of ^177^Lu-labeled RM1 or AMBA was monitored by a reduction in percent HPLC peak area with time. Half-lives of in vitro acetate buffer and serum degradation for each imaging pharmaceutical were calculated by fitting the percent peak remaining versus time to a first-order kinetics model (Equations (2) and (3)) [44] using Excel or Graph Pad Prism version 5.03 software (Graph Pad Software, Inc., La Jolla, CA, USA).

P_t_ = P_0_ exp (−k × t); (2)

t_1/2_ = 0.693/k; (3)

where P_t_ is the percent peak area at time t, P_o_ is the percent peak area initially, t is the time elapsed, k is the first-order rate constant, and t_1/2_ is the half-life of degradation reaction. All degradation kinetics experiments were conducted in triplicate, and average (n = 3) values (standard deviation given in the parenthesis) of half-life are reported. 

## 4. Conclusions 

This study demonstrates that (1) low-radioconcentration samples of ^177^Lu-labeled RM1 are stable for several days upon storage at room temperature and 2–8 °C; (2) the samples of ^177^Lu-labeled RM1 with high radioconcentration degrade much faster than low-radioconcentration samples upon storage at 2–8 °C; (3) stabilizers, ascorbic acid and gentisic acid, improve the stability of ^177^Lu-labeled RM1, with gentisic acid being more efficient than ascorbic acid as a stabilizer; (4) the degradation kinetics of ^177^Lu-labeled AMBA and RM1 follow the order (fastest to slowest): mouse > canine > human sera; and (5) the ^177^Lu-labeled RM1 antagonist is slower to degrade in mouse, canine, and human sera than the ^177^Lu-labeled AMBA agonist, further suggesting that an antagonist is a more promising candidate than agonist for PET imaging and therapy in prostate cancer patients. 

## Figures and Tables

**Table 1:** Summary of half-life of degradation (t_1/2_, h) of low and high-radioconcentration samples of ^177^Lu-Labeled RM1 in acetate buffer stored at room temperature and 2–8 °C.

Sample and Storage Condition	Stabilizer	Half-Life, h *
Low Radioconcentration, Room Temperature	None	164.3 (3.6)
Low Radioconcentration, 2–8 °C	None	195.6 (3.6)
High Radioconcentration, 2–8 °C	None	8.5 (0.2)
High Radioconcentration, 2–8 °C	Ascorbic Acid	39.2 (0.6)
High Radioconcentration, 2–8 °C	Gentisic Acid	142.5 (1.4)

* Average (with standard deviation in the parenthesis for n = 3) half-life.

**Table 2:** Summary of half-life of degradation (h) of ^177^Lu-Labeled RM1 and AMBA in acetate buffer, mouse, canine, and human sera.

Medium	RM1	AMBA
Acetate buffer, Room Temperature	164.3 (3.6)	87 (3.9)
Acetate buffer, 2–8 °C	195.6 (3.6)	127.7 (2.1)
Mouse Serum, 37 °C	16.4 (0.6)	3.1 **
Canine Serum, 37 °C	18.9 (0.8)	10.1 (0.5)
Human Serum, 37 °C	141 (5)	38.8 **

Average (with standard deviation in the parenthesis for n = 3) half-life of degradation, ** References [23,24].

## Supplementary Materials

The supplementary materials are available online.
