# Supplementary material for: Stability Evaluation and Stabilization of a Gastrin-Releasing Peptide Receptor (GRPR) Targeting Imaging Pharmaceutical
Source: Molecules. 2019 Aug 8;24(16):2878. doi: 10.3390/molecules24162878 (PMC6720803; doi:10.3390/molecules24162878)
Supplement: Supplementary file 1 [file molecules-24-02878-s001.pdf]

Stability Evaluation and Stabilization of a Gastrin-Releasing Peptide Receptor (GRPR) Targeting Imaging Pharmaceutical

Arijit Ghosh, Karen Woolum, Shankaran Kothandaraman, Michael F. Tweedle, Krishan Kumar *

Laboratory for Translational Research in Imaging Pharmaceuticals, Wright Center of Innovation for Biomedical Imaging, Department of Radiology, The Ohio State University, Columbus, Ohio 43212, USA

* Correspondence: Krishan.Kumar@osumc.edu

Supplementary Information

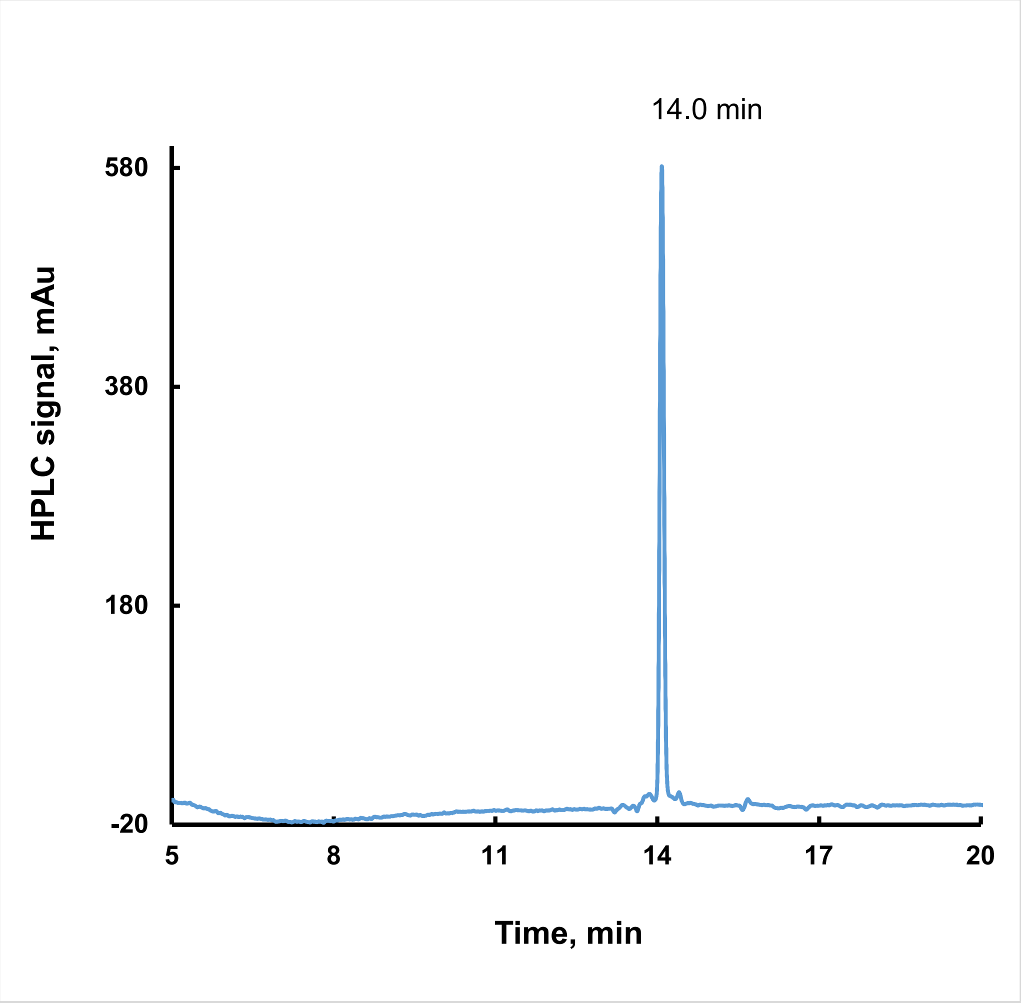

Figure S1: HPLC chromatogram of RM1.

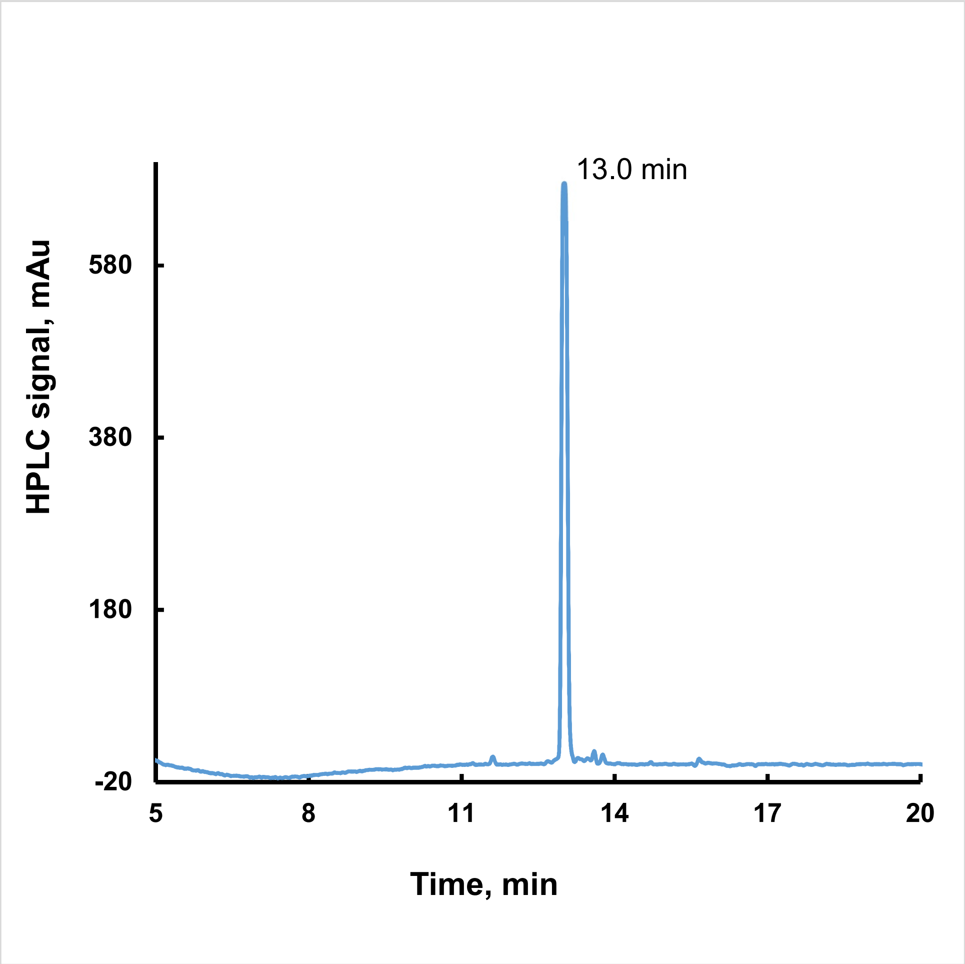

Figure S2: HPLC chromatogram of AMBA

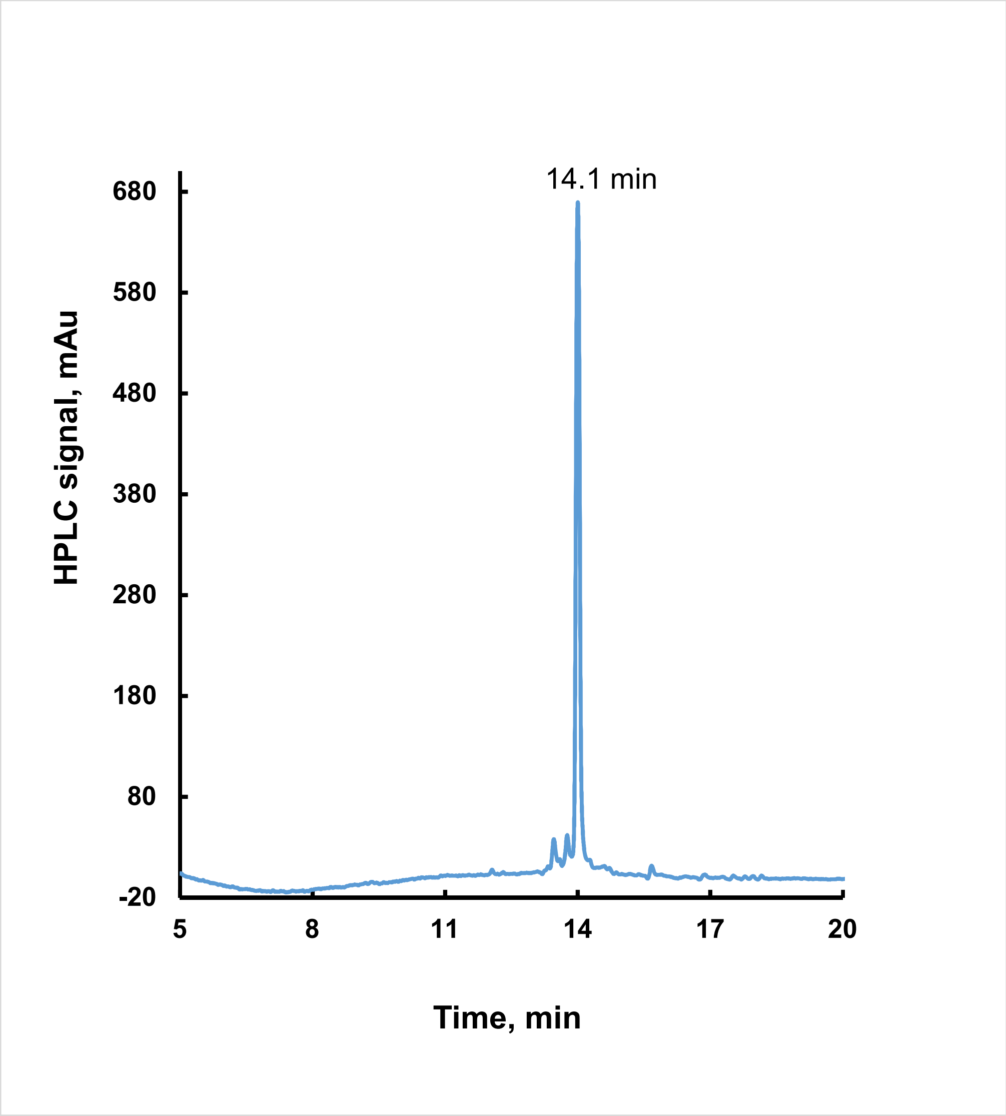

Figure S3: HPLC chromatogram of Lu-RM1.

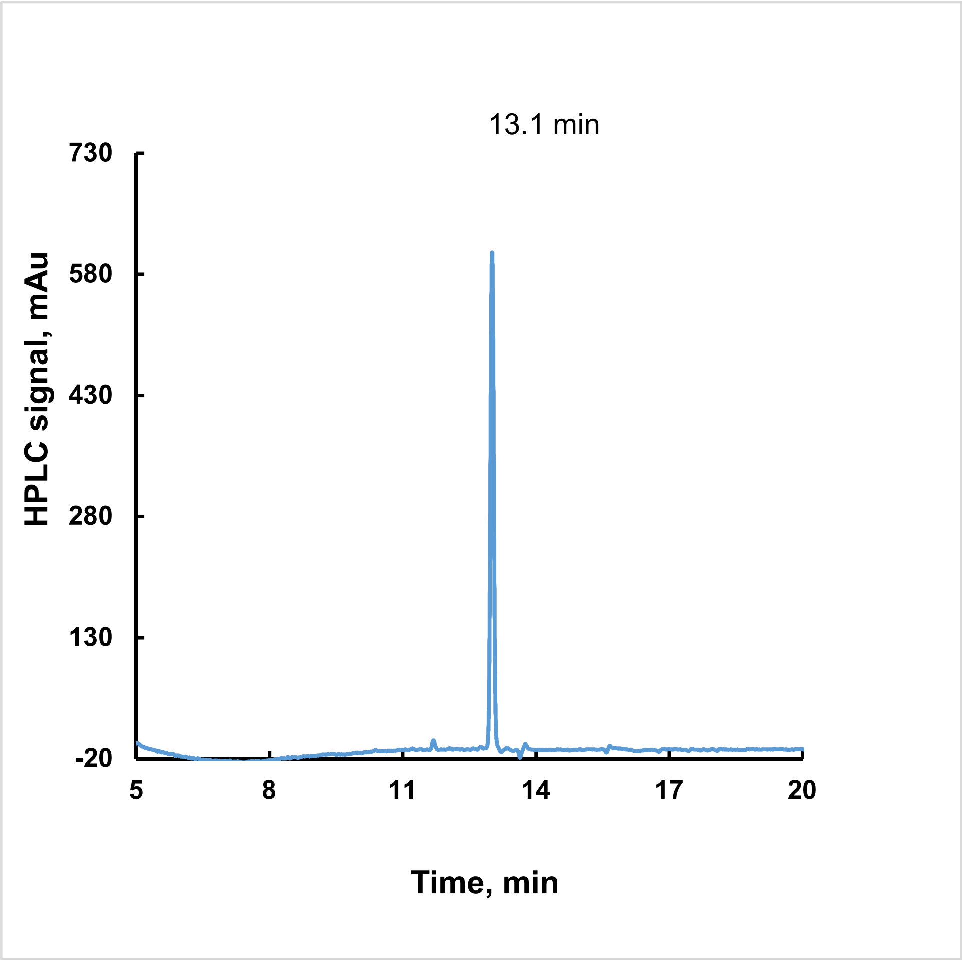

Figure S4: HPLC chromatogram of Lu-AMBA.

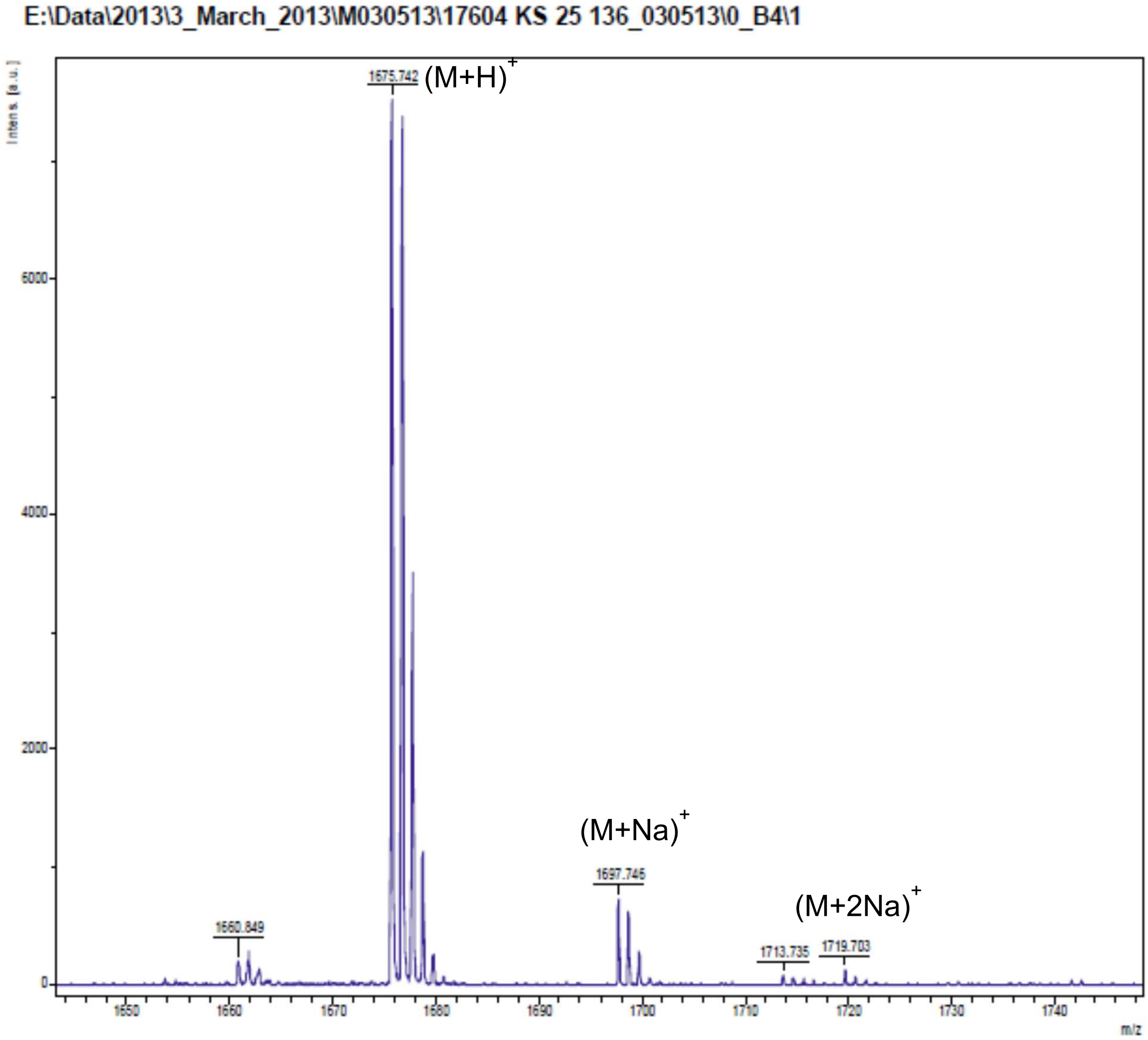

Figure S5: MALDI-TOF Mass spectrum of RM1

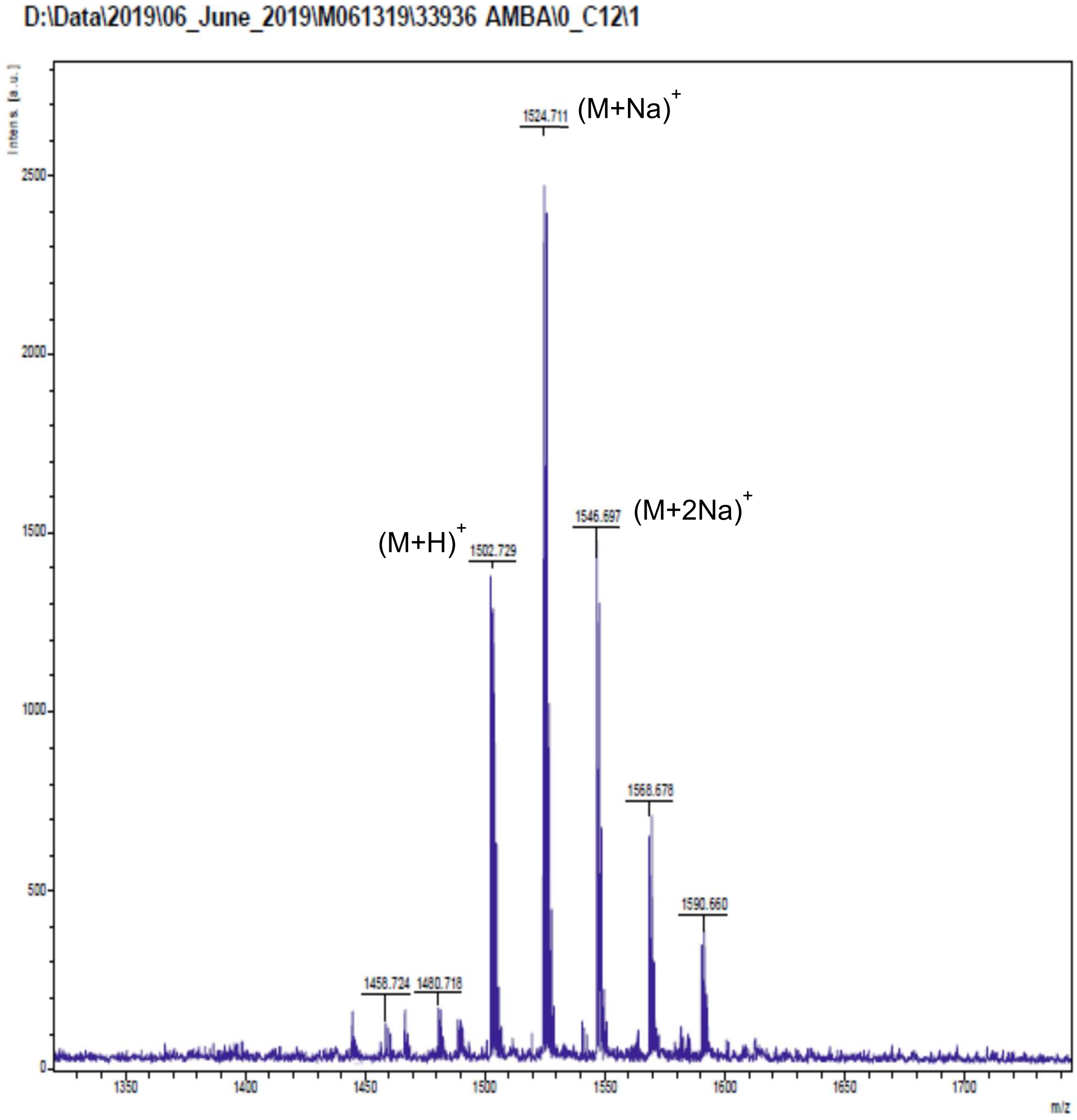

Figure S6: MALDI-TOF Mass spectrum of AMBA

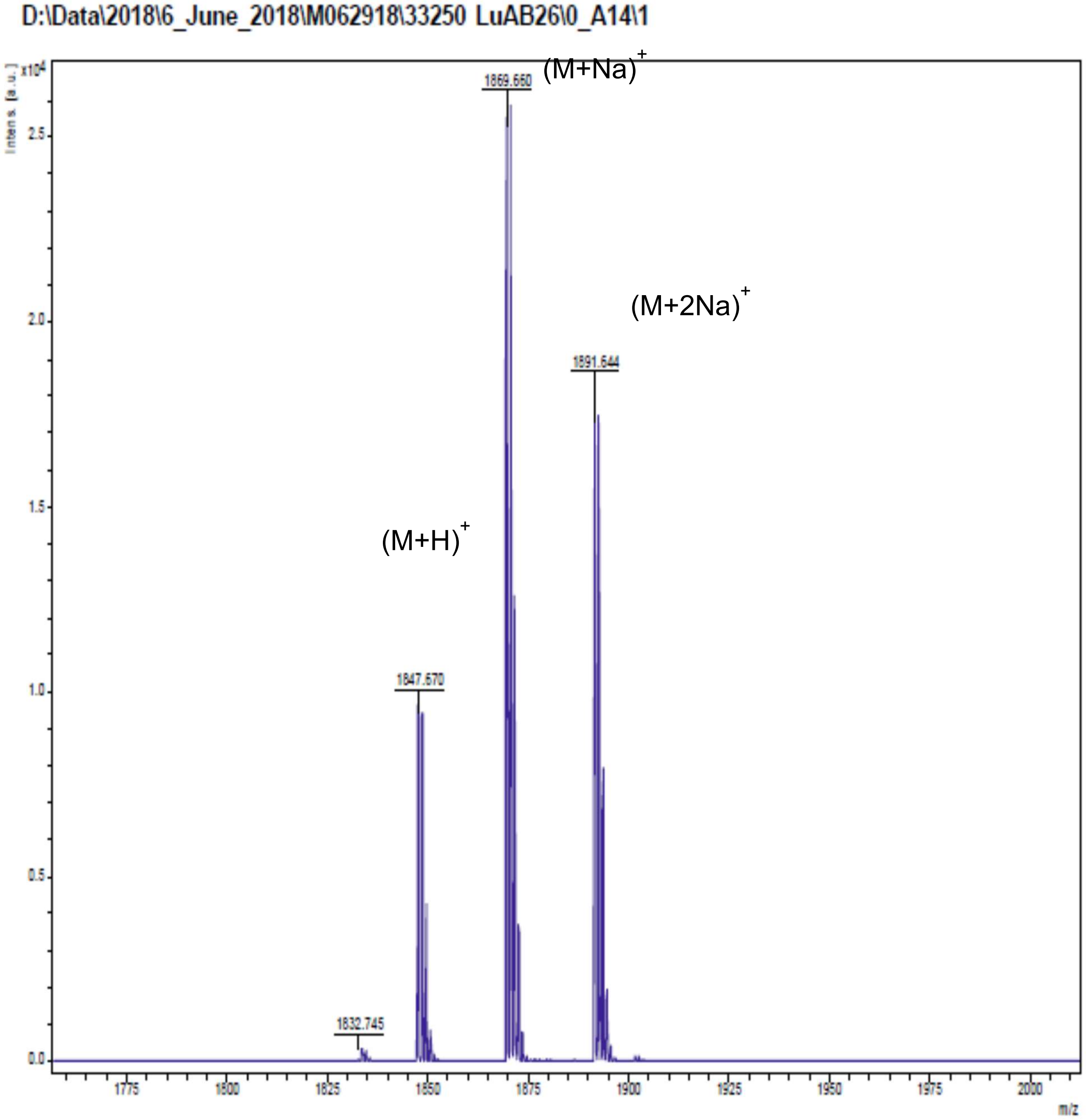

Figure S7: MALDI-TOF Mass spectrum of Lu-RM1

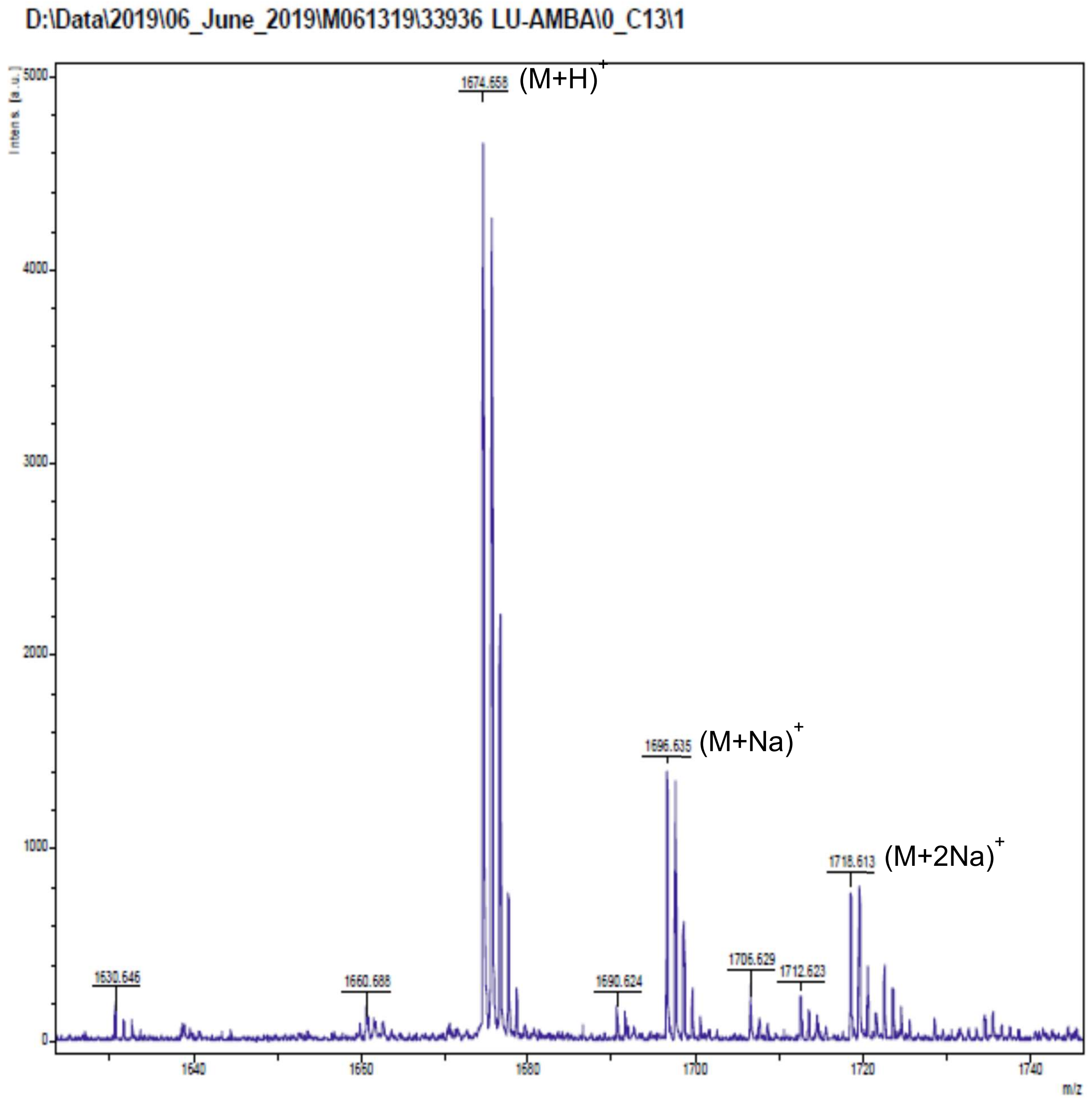

Figure S8: MALDI-TOF Mass spectrum of Lu-AMBA.

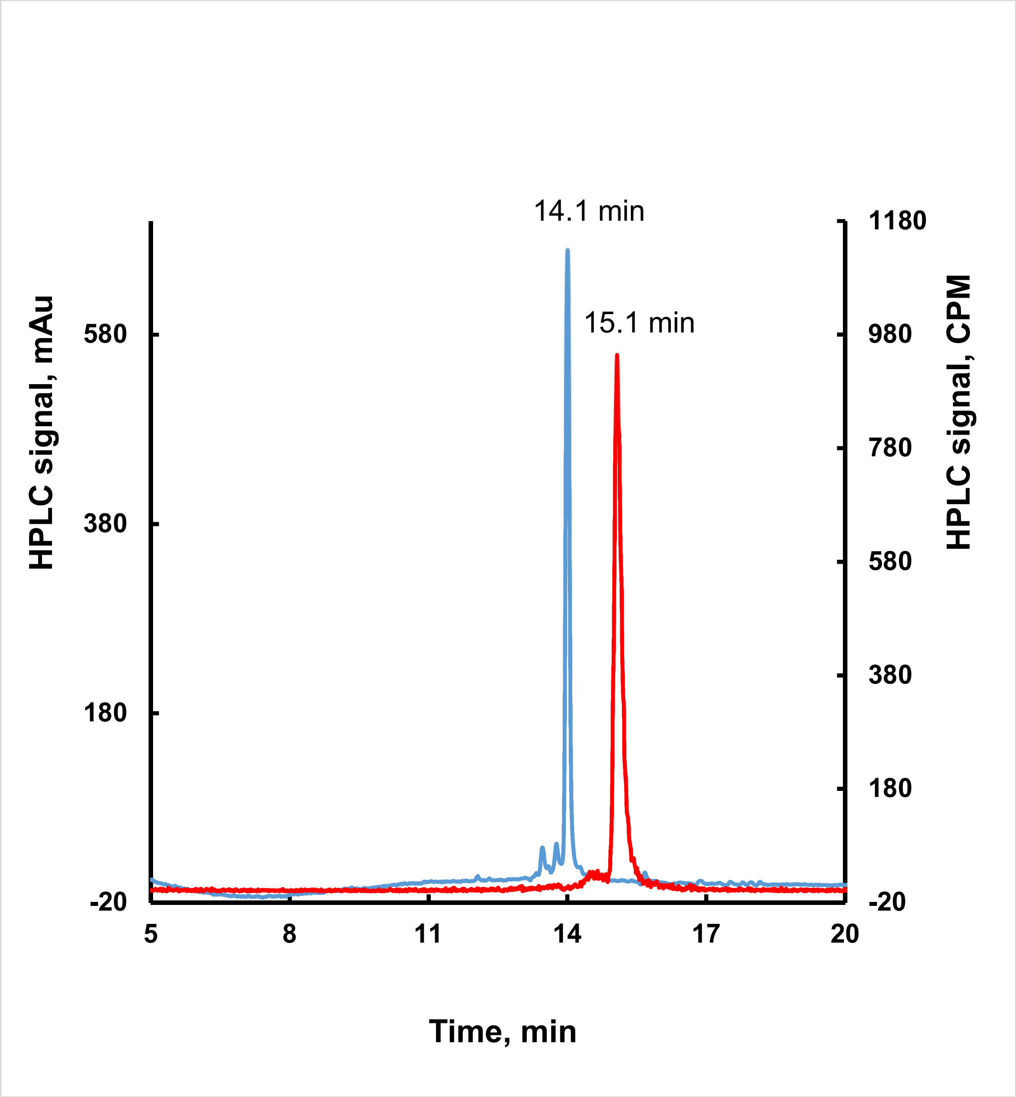

Figure S9: HPLC chromatograms of Lu-RM1 (Blue) and ^{177}Lu-RM1 (Red).

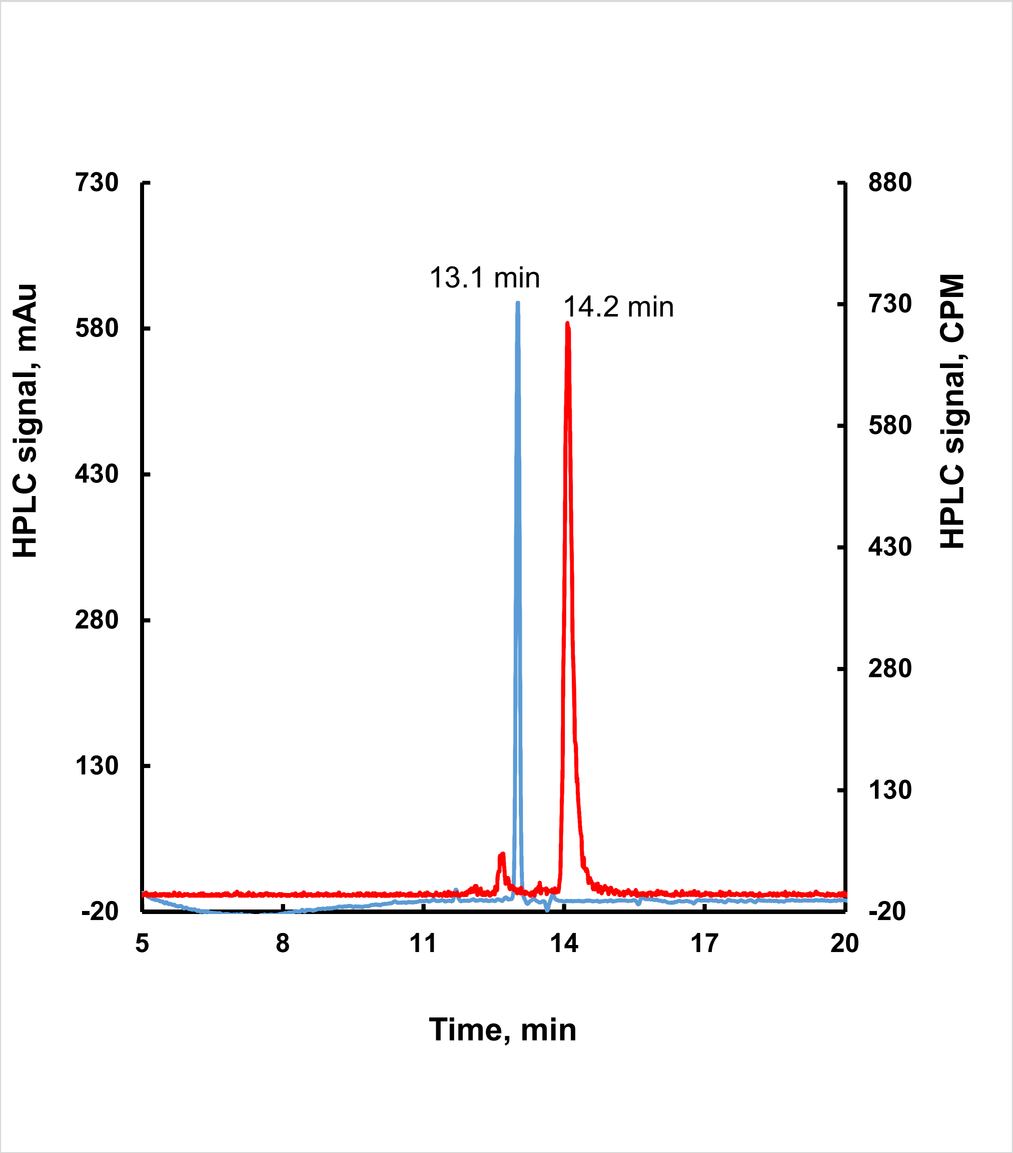

Figure S10: HPLC chromatograms of Lu-AMBA (Blue) and ^{177}Lu-AMBA (Red).
